# Modified Atmosphere Packaging Delays Senescence and Chlorophyll Degradation by Enhancing Antioxidant Capacity in Postharvest Broccoli

**DOI:** 10.3390/foods15132251

**Published:** 2026-06-23

**Authors:** Jingyu Xu, Lanying He, Letian Lin, Tianwen Liu, Baisi Tang, Honghui Luo, Hua Huang

**Affiliations:** 1 College of Horticulture and Landscape Architecture, Zhongkai University of Agriculture and Engineering, Guangzhou 510225, Chinaletianlin.edu@gmail.com (L.L.); 18011834905@163.com (B.T.); 2 Institute of Fruit Tree Research, Guangdong Academy of Agricultural Sciences; Key Laboratory of South Subtropical Fruit Biology and Genetic Resource Utilization, Ministry of Agriculture and Rural Affairs; Guangdong Provincial Key Laboratory of Science and Technology Research on Fruit Tree, Guangzhou 510640, China; 3 Guangdong Provincial Key Laboratory of Biotechnology for Plant Development, School of Life Sciences, South China Normal University, Guangzhou 510631, China

**Keywords:** broccoli, modified atmosphere packaging (MAP), commercial quality, chlorophyll degradation, reactive oxygen species (ROS), antioxidant capacity

## Abstract

Fresh broccoli is highly perishable, exhibiting rapid yellowing and quality deterioration with a short shelf life. In this study, we investigated the effects of nanomaterial-modified atmosphere packaging (MAP) bags with different thicknesses, designated as 2.5C (25 μm) and 4C (40 μm), on the physiological and biochemical changes in broccoli were evaluated during storage at 20 ± 1 °C for 8 days. Results showed that both MAP treatments remarkably delayed floret senescence by inhibiting the rapid color transition from green to yellow, as indicated by alterations in L*, a*, b*, and hue angle values, as well as by suppressing chlorophyll degradation. The 2.5C treatment exhibited a more pronounced effect during storage. MAP treatments helped maintain commercial quality by preserving total phenols and vitamin C (Vc) content, retaining stem firmness and surface glossiness, regulating post-opening respiration rate and reducing water loss. MAP treatments also effectively suppressed the accumulation of superoxide anion (O_2_•^−^) and hydrogen peroxide (H_2_O_2_). Furthermore, MAP treatments enhanced free radical scavenging capacity, as demonstrated by DPPH and ABTS assays and the O_2_•^−^ scavenging rate in broccoli. These results indicate that MAP treatment with an appropriate thickness (e.g., 2.5C) effectively inhibits excessive ROS production and enhances antioxidant capacity, thereby delaying floret chlorophyll degradation and senescence. This study provides a foundation for developing effective and green preservation strategies using physical MAP treatments for fresh broccoli.

## 1. Introduction

Broccoli (*Brassica oleracea* var. *italica*) is a biennial plant belonging to the family Brassicaceae. It originated in the Mediterranean region and is now widely planted and consumed [1,2]. The flowering head contains high nutritional value, including vitamins, minerals, dietary fiber, and bioactive compounds, which provide high health benefits of broccoli [3,4]. However, broccoli is highly susceptible to senescence after harvest, exhibiting symptoms such as chlorophyll degradation and quality deterioration [5]. This rapid senescence is mainly attributed to disruptions in the supply of hormones, nutrients, and energy, leading to an imbalance in source–sink transport and thereby triggering senescence [6]. Therefore, appropriate postharvest preservation methods are of great importance for extending the storage life of broccoli and enhancing its commercial value.

Postharvest treatments of broccoli have been addressed using various chemical or physical methods to prevent the rapid deterioration of broccoli during storage [7]. For instance, exogenous treatments with sodium benzoate [8], essential oil [9], melatonin [10], and folic acid [11] influenced chlorophyll catabolism and antioxidant defense to inhibit broccoli senescence. Application of 1-MCP efficiently depressed the loss of green color and chlorophyll degradation under low temperature [12]. Physical techniques such as LED green light [13] and controlled atmosphere [12] inhibited the decrease in phenolic and glucosinolate contents, thereby delaying floret senescence. However, with the growing demands for safety, efficiency, and cost-effectiveness in postharvest strategies, it remains necessary to develop novel techniques to delay the senescence of fresh produce, including broccoli.

The development and improvement of modified atmosphere packaging (MAP) represents an advanced preservation technology for food and horticultural produce [14]. MAP primarily functions by reducing oxygen concentration and increasing carbon dioxide concentration to establish gas homeostasis within the package. This helps to suppress respiration, ethylene production, and the excessive production of reactive oxygen species (ROS), thereby modulating physiological and biochemical processes [15]. A low-oxygen environment can directly limit aerobic respiration, reduce nutrient loss, and delay tissue senescence in fruits and vegetables [14]. It has been demonstrated that MAP treatment suppresses chloroplast dismantling and mitochondrial oxidative damage in Chinese flowering cabbage [16,17]. Additionally, MAP treatment could also inhibit cell wall metabolism and alleviate stem lignification in bamboo shoots during storage [18]. The above-reported treatments on Chinese flowering cabbage and bamboo shoots were adjusted with 30 μm MAP, effectively delaying senescence during storage. Recent advances in metal–organic frameworks (MOFs) have enabled their potential use as biomaterials for skin therapy and as nanocomposites to modify film permeability [19,20]. The modified packaging films or bags with MOF-nanomaterials have been shown with the efficiency of delaying senescence and quality loss in fruits, vegetables, and other edible foods [19,21]. However, package bags of different thicknesses typically exhibit different gas permeability, which affects the internal atmosphere [14,16]. The efficiency of MAP films or bags with different thicknesses for postharvest applications has not yet been evaluated. Therefore, the continued development of MAP techniques in an appropriate manner i.e., thickness, may greatly advance green preservation strategies for horticultural produce.

The present study aimed to investigate the effect of different thicknesses of MAP on the senescence of broccoli during storage. The changes in color, commercial rate, chlorophyll content, and ROS accumulation in broccoli florets were characterized. Quality traits such as vitamin C content, water status, stem hardness, and glossiness were also evaluated. Antioxidant capacity, including the O_2_•^−^ clearance rate as well as DPPH and ABTS radical scavenging activities, was analyzed. The application of MAP enhanced antioxidant capacity and inhibited chlorophyll degradation, providing an appropriate strategy for broccoli preservation.

## 2. Materials and Methods

### 2.1. Materials and Treatments

Broccoli with green floret head was obtained from a local vegetable field in Guangzhou, China. The fresh harvest broccoli was transported to the laboratory immediately. Samples with uniform head size, fine visual appearance, consistent green color, and free from mechanical damage were selected for the experiment. The selected florets were randomly divided into three groups. One group of samples were packed with market polyethylene bags at thicknesses of 30 μm (CO_2_ permeability, 2256.6 cm^3^ m^−2^ d^−1^ atm^−1^; O_2_ permeability, 1344.8 cm^3^ m^−2^ d^−1^ atm^−1^) as control, one group samples were packed with polyethylene bags modified with nanomaterials at thicknesses of 25 μm (2.5C, CO_2_ permeability, 24,700 cm^3^ m^−2^ d^−1^ atm^−1^; O_2_ permeability, 5420 cm^3^ m^−2^ d^−1^ atm^−1^), the third group samples were packed with polyethylene bags modified with nanomaterials at thicknesses of 40 μm (4 C, CO_2_ permeability, 13,200 cm^3^ m^−2^ d^−1^ atm^−1^; O_2_ permeability, 3040 cm^3^ m^−2^ d^−1^ atm^−1^). The nanomaterial-modified bags were fabricated via the loading of nano-scale mica and diatomaceous earth as functional fillers. The mica platelets help to create tortuous diffusion pathways, and the porous diatomaceous earth provides gas-exchange channels, which together enables the modulation of gas permeability of CO_2_ and O_2_. The packaging bags were used without any additional disinfection treatment and were heat-sealed immediately after the broccoli samples were placed inside. Three florets were packed in one bag, and three bags were prepared for each storage time point. Samples were stored at ambient room conditions at 20 ± 1 °C and RH 85 ± 5%. The relatively high humidity was mainly due to the naturally humid ambient conditions in Guangzhou, where the experiment was conducted. The appearance color, commercially acceptable rate were evaluated at 0, 2, 4, 6, and 8 d during storage. After each sampling evaluation, the broccoli floret tissues were immediately frozen in liquid nitrogen and stored at −80 °C. Before biochemical analysis, the frozen tissues were ground into a fine powder under liquid nitrogen. The required amounts of frozen powdered tissue were then weighed for the determination of superoxide anion content, hydrogen peroxide content, superoxide anion scavenging capacity, and DPPH and ABTS radical scavenging capacities.

### 2.2. Appearance Imaging, Commercial Acceptability, Respiration Rate, Weight Loss, and Stem Firmness Assay

Images of sample appearance were captured using a SONY Alpha 6000 camera (Sony, Tokyo, Japan) equipped with a SIGMA 56 mm f/1.4 DC DN Contemporary lens (Sigma, Kawasaki, Japan). Image stitching and layout arrangement were completed using Adobe Photoshop 2020.

Commercial acceptability and weight loss of broccoli were determined according to the previous method with slight modifications [22]. Before evaluation, broccoli samples from different packaging treatments were randomly coded to blind the evaluator to the treatment information. For each treatment and sampling time, three biological replicates were visually assessed. The commercially acceptable rate was calculated as the percentage of the non-yellowed and non-wilted surface area relative to the total visible surface area of each broccoli head, and the mean value was used for statistical analysis. The respiration rate of broccoli after MAP storage under air conditions was determined using the open-bag followed by static, sealed method. Broccoli stored under MAP was removed, and 160 ± 20 g of broccoli florets were cut from each sample and immediately placed in a sealed glass jar. The CO_2_ concentration inside the jar was continuously recorded for 5 min using a T7001 gas detector (Telaire, Goleta, CA, USA). The mass of each sample was recorded before measurement. In addition, the density of the broccoli was determined using the water-displacement method, and the volume of each sample was subsequently calculated. The volume occupied by the measuring instrument inside the jar was also subtracted when calculating the effective headspace volume. The respiration rate was calculated based on the slope of the CO_2_ concentration increase over time, the effective gas volume of the sealed jar, and the sample mass. Results are expressed as mg CO_2_ kg^−1^ h^−1^. Weight loss was calculated according to the change in sample mass before and after storage. Main stem firmness was measured using a fruit firmness tester GY-4 (Shandong Lanjing Electronic Technology Co., Ltd., Weifang, China) equipped with a cylindrical metal probe with a diameter of 3.5 mm, and the results were expressed in N.

### 2.3. Determination of Color Parameters and Glossiness

The color parameters L*, a*, and b* of broccoli heads were measured using a spectrophotometric colorimeter PS2020 (3nh, Shenzhen, China). Measurements were conducted according to the standards recommended by the International Commission on Illumination (CIE, 2004), using a D65 standard illuminant and a 10° standard observer angle, and the hue angle (h°) was calculated.

According to the method of Zhang [23], the surface glossiness of the main stem of broccoli was measured using a gloss meter NHG60 (3nh, Shenzhen, China). During measurement, the sample surface was illuminated at an incident angle of 60°, and the reflected light intensity was recorded. The results were expressed in gloss units (GU).

### 2.4. Determination of Chlorophyll and Total Phenolic Contents

Chlorophyll and total phenolic contents were determined according to method of Huang [24] with minor modifications. For chlorophyll determination, broccoli bud tissue was homogenized and extracted with 80% (*v*/*v*) acetone in darkness overnight. After filtration, the absorbance was measured at 663 and 645 nm. Chlorophyll content was calculated and expressed as g kg^−1^ on basis of fresh weight.

Total phenolic content was determined using a modified Folin–Ciocalteu colorimetric method. Broccoli floret tissues were homogenized and extracted with 60% ethanol. Gallic acid was used to establish the standard calibration curve. The sample extract (0.25 mL) was mixed with 1 mL of 10% Folin–Ciocalteu reagent and incubated for 6 min. Subsequently, 3 mL of 7.5% sodium carbonate solution was added, and the mixture was diluted to 10 mL with distilled water. After incubation in darkness for 2 h, the absorbance was measured at 765 nm. Total phenolic content was expressed as mg gallic acid equivalents g^−1^ on the basis of fresh weight.

### 2.5. Determination of Vitamin C Content

Vitamin C (Vc) content was determined according to the reported method with slight modifications [25]. Broccoli floret tissue was ground and homogenized with distilled water to prepare the sample extract. Then, 1 mL of the sample extract was mixed with 8 mL of oxalic acid–EDTA solution, 1 mL of metaphosphoric acid–acetic acid solution, 2 mL of diluted sulfuric acid, and 4 mL of ammonium molybdate solution. The mixture was vortexed thoroughly and allowed to react for 16 min. The absorbance was then measured at 705 nm. Distilled water was used as the blank control. Each treatment was measured in triplicate, and Vc content was calculated from the standard curve.

### 2.6. Determination of Superoxide Anion and Hydrogen Peroxide Content

The contents of superoxide anion (O_2_•^−^) and hydrogen peroxide (H_2_O_2_) in broccoli were determined using Solarbio assay kits (Solarbio, Beijing, China). Approximately 0.1 g powdered samples were mixed with 1.0 mL of the extraction solution, and homogenized on an ice bath. After centrifuged at 4 °C, the supernatant was collected for analysis. Superoxide anion content was determined using a superoxide anion assay kit (Solarbio, Beijing, China; BC1290), and absorbance was recorded at 530 nm. Hydrogen peroxide content was determined using a hydrogen peroxide assay kit (Solarbio, Beijing, China; BC3590), and absorbance was recorded at 415 nm. The results were calculated and expressed as mmol kg^−1^ on basis of fresh weight.

### 2.7. Determination of Superoxide Anion Scavenging Capacity

The superoxide anion scavenging capacity of broccoli samples was determined using a superoxide anion scavenging capacity assay kit (Solarbio, Beijing, China; BC1410). Briefly, 0.1 g of broccoli floret tissues were weighed and mixed with 1.0 mL of extraction solution. After homogenization, mixtures were centrifuged at 4 °C and 10,000× *g* for 10 min, and the supernatant was collected for analysis. The reaction system was prepared, followed by incubation at 25 °C for 1 min and 37 °C for 30 min. Absorbance was measured at 530 nm. The superoxide anion scavenging rate was calculated based on the absorbance values of the blank and sample tubes, and the results were expressed as percentage (%).

### 2.8. Assay of ABTS and DPPH Radical Scavenging Capacities

For determination of ABTS and DPPH radical scavenging capacity, a 0.15 g portion of broccoli floret tissue was homogenized with 1.5 mL absolute ethanol and 1.5 mL 80% (*v*/*v*) ethanol. After extraction for 20 min on ice bath, the samples were centrifuged, and the supernatants were collected for further analysis.

For assay ABTS radical scavenging capacity, the sample extracts were mixed with appropriately diluted ABTS working solution and reacted for 30 min in darkness as the sample group (A). Absolute ethanol was used instead of the sample extracts as the blank group (A_0_). Absorbance values were measured at 734 nm. The ABTS radical scavenging rate was calculated as (A_0_ − A)/A_0_ × 100, and result was expressed as percentage %.

For assay the DPPH radical scavenging capacity, the appropriately diluted sample extract was mixed with DPPH working solution and allowed to stand at room temperature for 30 min as the sample group (A). The blank control group (A_0_) was prepared using 80% (*v*/*v*) ethanol instead of the sample extract, while the background control group (B) was prepared using 80% ethanol instead of the DPPH working solution. Absorbance values were measured at 517 nm. DPPH radical scavenging rate was calculated as [1 − (A − B)/A0] × 100, and results were expressed as percentage %.

### 2.9. Statistical Analysis

Statistical analysis was performed using SPSS software (Version 27.0.1, IBM, Armonk, NY, USA). All experiments were independently repeated at least three times (*n* ≥ 3). Statistical significance was determined by two-way analysis of variance (ANOVA), followed by Tukey’s HSD multiple comparison test. Differences were considered significant at *p* < 0.05. Figures were prepared using Origin software 17.0 (OriginLab, Northampton, MA, USA).

## 3. Results and Discussion

### 3.1. Effects of MAP on the Color Parameters of Broccoli During Storage

**Figure 1:**
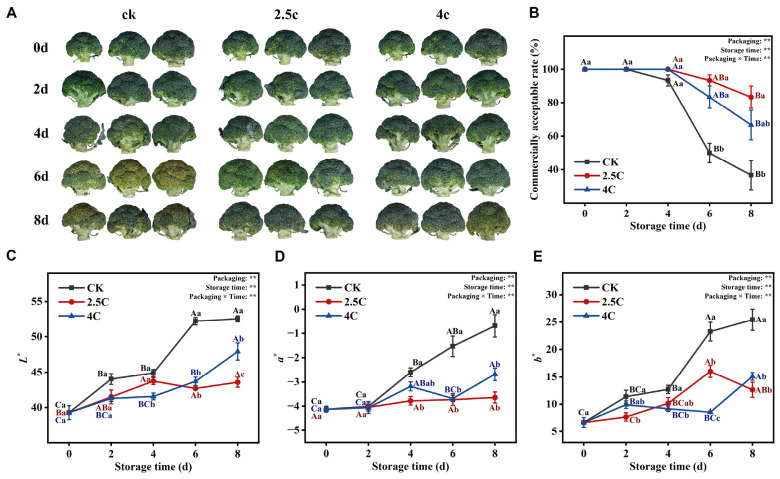
**Effects of MAP treatments on senescence of broccoli and changes in color of broccoli during storage.** (**A**) Visual changes of broccoli; (**B**) commercially acceptable rate of broccoli during storage; (**C**) L* value, (**D**) a* value, and (**E**) b* value of broccoli. MAP with 25 μm and 40 μm thickness were applied. Data are presented as mean ± standard deviation. Statistical significance was determined by two-way ANOVA followed by Tukey’s HSD test. Different lowercase letters indicate significant differences among packaging treatments at the same storage time, while different uppercase letters indicate significant differences among storage times within the same packaging treatment (*p* < 0.05). Two-way ANOVA showed significant effects of storage time, packaging treatment, and their interaction on the measured index. Asterisks indicate significant differences, with “**” indicating *p* < 0.01.

Broccoli is highly perishable, prone to yellowing, water loss, softening, and nutrient degradation, which together cause a rapid decline in commercial value [26,27,28]. As shown in Figure 1A, both MAP treatments alleviated floret yellowing process compared with the control, with the 2.5C packages demonstrating greater efficacy during storage. Commercial acceptability gradually decreased in all groups over extended storage, but MAP treatment significantly slowed this decline. The acceptability of the control group began to decline at 4 d and fell to 36.7% by 8 d, whereas the 2.5C and 4C groups retained values of 83.3% and 66.7%, respectively (Figure 1B). The decline in commercial acceptability was consistent with the progressive yellowing observed in the photographs and the corresponding changes in the color parameters. In particular, the marked decrease in commercial acceptability in the control group during the later storage period coincided with greater changes in L*, a*, and b*, indicating more pronounced visual-quality deterioration. In contrast, the relatively higher commercial acceptability of the 2.5C treatment was associated with its greener visual appearance and smaller overall changes in the measured color parameters.

MAP has been widely reported to extend the shelf life of Chinese flowering cabbage and other vegetables by retarding green color loss and chlorophyll degradation [18]. In the control group, the color parameter L* increased markedly after 6 d, indicating brightness shifts and color loss, while both MAP-treated samples showed much slower changes (Figure 1C). The negative a* value rose progressively, reflecting a gradual loss of green color and onset of yellowing with prolonged storage (Figure 1D). Similar to change in L* value, the b* values increased remarkably after 6 d in control, and both MAP treatments slowed down this trend (Figure 1E). Although statistically significant differences in a* and b* were detected among treatments at some sampling times, including Day 4, the numerical differences were relatively small. Regarding the changes in these color parameters, which could indicate the overall color modifications [29,30], MAP with 2.5C showed the most obvious inhibitory effect on delaying the yellowing process, followed by the 4C treatment. These results indicate that appropriate MAP treatment can effectively delay postharvest yellowing of broccoli during storage.

### 3.2. Effects of MAP Treatments on Respiration Rate Characteristics

**Figure 2:**
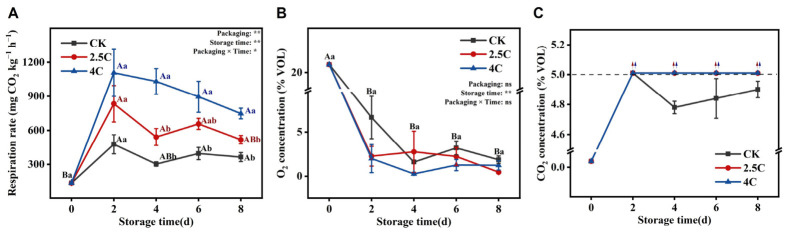
**Effects of MAP treatments on gas composition during storage.** (**A**) respiration rate of broccoli after MAP storage, measured in sealed glass jars immediately after the MAP packages were opened; (**B**) Oxygen concentration in modified atmosphere packaging; (**C**) carbon dioxide concentration in modified atmosphere packaging. The dashed line indicates the upper measurement limit of the instrument. Data points marked with arrows exceeded this limit and should be interpreted as values greater than the measurable range, rather than exact measurements. MAP with 25 μm and 40 μm thickness were applied. Data are presented as mean ± standard deviation. Statistical significance was determined by two-way ANOVA followed by Tukey’s HSD test. Different lowercase letters indicate significant differences among packaging treatments at the same storage time, while different uppercase letters indicate significant differences among storage times within the same packaging treatment (*p* < 0.05). Two-way ANOVA showed significant effects of storage time, packaging treatment, and their interaction on the measured index. Asterisks indicate significant differences, with “*” indicating *p* < 0.05 and “**” indicating *p* < 0.01; “ns” indicates no significant difference (*p* > 0.05).

Although the CK group was also packaged, it was packed in ordinary household fresh-keeping bags, whereas the MAP treatments used bags with different gas-permeability properties. As shown in the Figure 2A, the respiration rate measured after package opening was higher in the MAP-treated samples than in CK, particularly at the early stage of storage. Although respiration declined thereafter, MAP treatments generally maintained higher post-opening respiration than CK, indicating a more pronounced respiratory rebound after exposure to ambient air.

It should be noted that the values shown in the figure represent the respiration rate measured after package opening, rather than the actual metabolic level under sealed in-package storage. During sealed storage, MAP can create an atmosphere with reduced O_2_ and elevated CO_2_ concentrations, which may contribute to respiration suppression, lower metabolic activity, and reduced substrate consumption in broccoli tissues [31,32]. Therefore, the in-package respiratory metabolism of the MAP-treated samples was likely lower than that of CK. After opening the packages, however, the modified atmosphere was rapidly disrupted. The re-entry of ambient O_2_ and the reduction of CO_2_ inhibition may have reactivated tissue respiration, resulting in a possible respiratory rebound [33,34,35]. Nevertheless, because respiration was not monitored under sealed in-package conditions, this explanation remains hypothetical and should be interpreted with caution.

As shown in Figure 2B,C, MAP treatments markedly altered the in-package gas composition during storage. Compared with CK, the MAP-treated groups showed relatively lower O_2_ accumulation and higher CO_2_ levels, indicating that the modified atmosphere was effectively established in the 2.5C and 4C treatments. The lower respiration rate observed in CK after package opening does not necessarily indicate better preservation [33]. In the early stage of storage, the sealed MAP environment, particularly in the 4C treatment with stronger sealing performance, may have imposed greater metabolic stress on broccoli tissues. Therefore, the relatively high respiration rate detected after package opening may be associated with a respiratory burst or rebound effect caused by the abrupt transition from the sealed modified atmosphere to ambient conditions, with this effect being more pronounced in the 4C treatment.

A true decline in tissue physiological activity was more likely to occur at the later storage stage, particularly after day 6, when the florets showed visible loss of green color. Therefore, the reduced respiration capacity in CK at the later stage may indicate decreased tissue activity rather than lower metabolic consumption [36]. Conversely, the higher post-opening respiration rate of the MAP-treated samples may suggest that the tissues retained higher physiological activity. Overall, MAP reduced the metabolic level of broccoli during sealed storage and helped delay senescence, whereas the elevated respiration rate observed after opening was mainly or at least partly caused by the abrupt change in gas composition. Therefore, it should not be simply interpreted as evidence that MAP promoted metabolic consumption during storage.

### 3.3. Effects of MAP Treatments on Changes in Chlorophyll and Phenol Content

**Figure 3:**
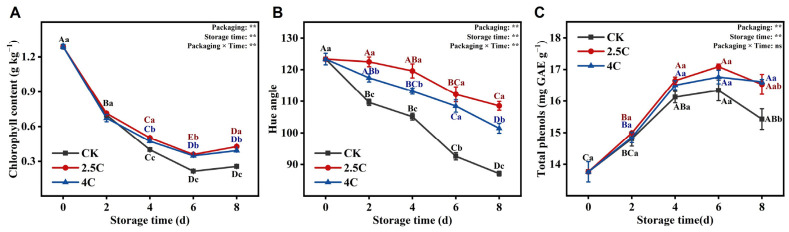
**Effects of different treatments on chlorophyll content, hue angle, and total phenolic content of broccoli during storage.** (**A**) Chlorophyll content, (**B**) hue angle, and (**C**) total phenolic content. MAP with 25 μm and 40 μm thickness were applied. Data are presented as mean ± standard deviation. Statistical significance was determined by two-way ANOVA followed by Tukey’s HSD test. Different lowercase letters indicate significant differences among packaging treatments at the same storage time, while different uppercase letters indicate significant differences among storage times within the same packaging treatment (*p* < 0.05). Two-way ANOVA showed significant effects of storage time, packaging treatment, and their interaction on the measured index. Asterisks indicate significant differences, with “**” indicating *p* < 0.01; “ns” indicates no significant difference (*p* > 0.05).

Chlorophyll degradation is strongly influenced by temperature in many plants [37,38]. Under storage conditions, chlorophyll content decreases rapidly as yellowing progresses, whereas low temperatures inhibit this process, resulting in higher chlorophyll retention [24,38]. As shown in Figure 3A, chlorophyll content in broccoli declined gradually across all treatments during storage. The decrease was slower in the 2.5C and 4C groups, with their chlorophyll contents being approximately 66.6% and 53.0% higher than that of the control, respectively. Overall, the control group showed a more pronounced decline in chlorophyll content, whereas the 2.5C and 4C groups maintained relatively higher chlorophyll levels during the later stages of storage.

Changes in hue angle values were consistent with the trends observed for chlorophyll content. Hue angle is an important color parameter used to indicate the green color status of plant surfaces [24,39]. The hue angle gradually decreased in all groups, indicating that broccoli florets transitioned from green to yellow-green. The hue angle declined most markedly from an initial value of about 123° to 87° in the control group, while both MAP-treated groups maintained values above 100° at 8 d (Figure 3B). In addition, the 2.5C treatment was more effective than 4C in delaying chlorophyll degradation. These results were highly consistent with the observed changes in commercial acceptability and color parameters.

As shown in Figure 3C, the total phenolic content initially increased and then slowly decreased during storage. This pattern suggests that broccoli may enhance its antioxidant capacity in the early storage stage by promoting phenolic compound accumulation. The 2.5C and 4C groups maintained relatively high phenolic levels, significantly higher than the control, with the 2.5C treatment showing a better effect. Phenol content is closely associated with antioxidant activity in fruits and vegetables [40,41]. Collectively, these results indicate that MAP treatment helps delay the depletion of total phenolics and preserves higher antioxidant potential.

### 3.4. Effects of MAP Treatments on Quality Traits and Texture Characteristics

**Figure 4:**
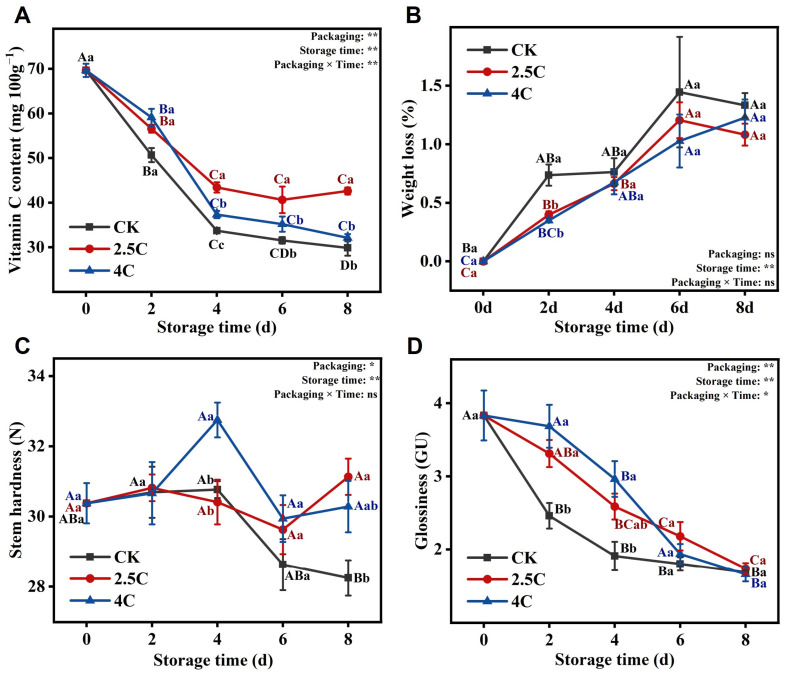
**Effects of different treatments on quality changes of broccoli during storage.** (**A**) vitamin C content, (**B**) weight loss (**C**) Stem glossiness, and (**D**) stem hardness. MAP with 25 μm and 40 μm thickness were applied. Data are presented as mean ± standard deviation. Statistical significance was determined by two-way ANOVA followed by Tukey’s HSD test. Different lowercase letters indicate significant differences among packaging treatments at the same storage time, while different uppercase letters indicate significant differences among storage times within the same packaging treatment (*p* < 0.05). Two-way ANOVA showed significant effects of storage time, packaging treatment, and their interaction on the measured index. Asterisks indicate significant differences, with “*” indicating *p* < 0.05 and “**” indicating *p* < 0.01; “ns” indicates no significant difference (*p* > 0.05).

As shown in Figure 4A, vitamin C content gradually decreased in all treatment groups with prolonged storage. The Vc content declined markedly by 57% in the control group at 8 d, while the 2.5C and 4C treatments inhibited Vc degradation, with decreases of 39% and 54%, respectively (Figure 4A). Vitamin C and total phenolics are important indicators of nutritional quality and antioxidant potential in broccoli [42,43]. In this study, Vc content continuously declined, whereas total phenolic content first increased and then decreased, suggesting that broccoli may enhance its antioxidant defense in the early postharvest stage by promoting phenolic compound accumulation [44]. MAP treatments, particularly 2.5C, remarkably delayed Vc degradation and the decline in total phenolic levels, indicating that MAP helps reduce antioxidant depletion and preserve nutritional quality [11].

Weight loss and tissue softening are important factors contributing to postharvest quality deterioration in broccoli [9,45]. Weight loss increased continuously with storage time across all groups. Both 2.5C and 4C treatments exhibited lower weight loss than the control group throughout storage (Figure 4B). This increasing weight-loss trend was accompanied by gradual changes in stem firmness and surface glossiness, particularly during the later storage period. It indicated that MAP treatment effectively inhibited water loss and alleviated wilting and quality deterioration caused by dehydration.

In addition to changes in florets, stem firmness showed an overall declining trend with gradual changes during storage (Figure 4C). In the control group, stem firmness changed slightly during the early storage period but declined clearly after 6 d. The 2.5C treatment maintained relatively high firmness throughout storage, while the 4C treatment showed a slight decline after 4 d. The decrease in firmness generally coincided with increasing weight loss, suggesting that progressive water loss and tissue deterioration may have contributed to reduced mechanical resistance. Nevertheless, because firmness can also be affected by cell-wall metabolism and tissue structure, the observed changes cannot be attributed to water loss alone.

Surface glossiness of the broccoli stem decreased continuously across all treatments (Figure 4D). In the control group, stem glossiness declined from approximately 3.83 to about 1.69 at 8 d, whereas both MAP treatments significantly delayed this decrease. Surface glossiness has been shown to be largely related to surface microstructures and epidermal cell morphology [46,47]. The decrease in glossiness, together with increasing weight loss, may reflect progressive surface dehydration, epidermal shrinkage, and structural deterioration.

It should be noted that surface glossiness and the L* value describe different optical properties. Glossiness mainly represents specular reflection from the sample surface, whereas L* represents the overall lightness of diffusely reflected light. Therefore, the higher L* value observed in the control group does not indicate higher surface glossiness or better freshness. In the control samples, the increase in L* occurred simultaneously with an increase in b*, a decrease in hue angle, greater weight loss, and lower stem glossiness. This combined pattern indicates that the higher L* value was more likely associated with progressive yellowing and surface lightening, whereas the reduction in glossiness was associated with water loss and surface deterioration.

Overall, MAP treatment may help reduce water loss, shrinkage, and surface damage, thereby preserving appearance glossiness. Accordingly, the results showed that MAP treatments reduced weight loss and, to some extent, maintained stem surface gloss and firmness, indicating that these treatments help alleviate moisture loss and structural damage. The 2.5C treatment exhibited a more stable effect on maintaining weight and firmness during the later storage stage, consistent with its higher commercial acceptability.

### 3.5. Effects of MAP Treatments on ROS Production and Antioxidant Activity Levels

**Figure 5:**
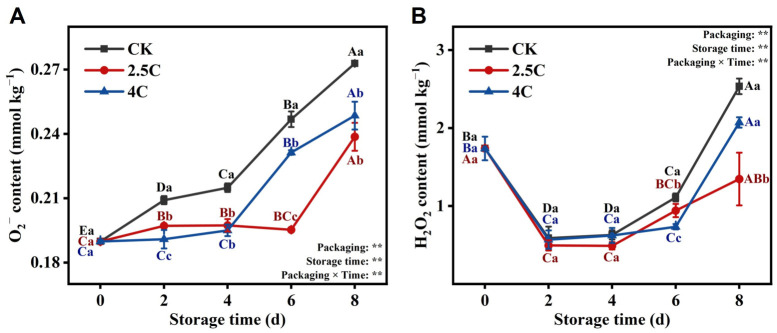
**Effects of different treatments on superoxide anion radical and hydrogen peroxide contents in broccoli during storage.** Changes in (**A**) O_2_•^−^ content and (**B**) H_2_O_2_ content of broccoli. MAP with 25 μm and 40 μm thickness were applied. Data are presented as mean ± standard deviation. Statistical significance was determined by two-way ANOVA followed by Tukey’s HSD test. Different lowercase letters indicate significant differences among packaging treatments at the same storage time, while different uppercase letters indicate significant differences among storage times within the same packaging treatment (*p* < 0.05). Two-way ANOVA showed significant effects of storage time, packaging treatment, and their interaction on the measured index. Asterisks indicate significant differences, with “**” indicating *p* < 0.01.

The level of ROS greatly influences cell metabolism in plant tissues [17,48]. In broccoli, the production of O_2_•^−^ and H_2_O_2_ generally increased during storage (Figure 5A). Compared with the control group, both the 2.5C and 4C treatments significantly suppressed the increases in O_2_•^−^ and H_2_O_2_ contents. Specifically, O_2_•^−^ content increased slowly and then rose sharply after 6 d in the 2.5C group and after 4 d in the 4C group (Figure 5A). H_2_O_2_ content showed a slight decline at the early stage and subsequently increased rapidly, with a much greater increase observed in the control group (Figure 5B). Previous studies have shown that various MAP treatments can inhibit excessive ROS production and delay senescence in Chinese flowering cabbage [17], bamboo shoots [18], and banana [49]. In the present study, the 2.5C treatment exhibited a more pronounced effect, effectively alleviating ROS accumulation and oxidative stress, thereby delaying senescence in broccoli.

**Figure 6:**
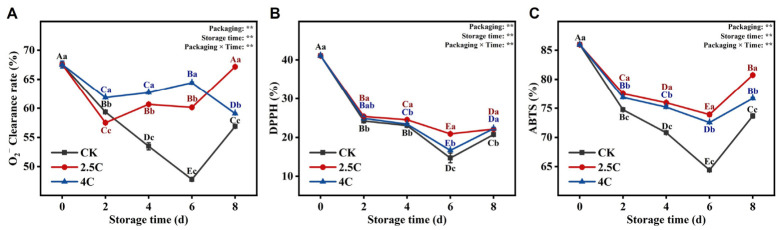
**Changes in antioxidant capacity of broccoli during storage.** (**A**) O_2_•^−^ clearance rate, (**B**) DPPH radical scavenging activity, and (**C**) ABTS radical scavenging activity of broccoli. MAP with 25 μm and 40 μm thickness were applied. Data are presented as mean ± standard deviation. Statistical significance was determined by two-way ANOVA followed by Tukey’s HSD test. Different lowercase letters indicate significant differences among packaging treatments at the same storage time, while different uppercase letters indicate significant differences among storage times within the same packaging treatment (*p* < 0.05). Two-way ANOVA showed significant effects of storage time, packaging treatment, and their interaction on the measured index. Asterisks indicate significant differences, with “**” indicating *p* < 0.01.

In addition to suppressing excessive ROS production, enhancing antioxidant activity is crucial for maintaining ROS homeostasis and cellular integrity [41,48]. As shown in Figure 6A, the O_2_•^−^ scavenging rate in the control group declined markedly after 6 d, indicating a significant loss of ROS elimination capacity. In contrast, both the 2.5C and 4C treatments effectively delayed this decline. The 4C treatment maintained a relatively high scavenging rate at 6 d, whereas the 2.5C treatment exhibited a higher rate through 8 d (Figure 6A). These results indicate that MAP treatment effectively preserves the ability of broccoli to scavenge superoxide anions, thereby reducing oxidative damage.

The DPPH radical scavenging activity, reflecting hydrogen- and electron-donating capacity, gradually decreased in broccoli during storage. Among all groups, the 2.5C treatment maintained higher DPPH scavenging capacity throughout the storage period (Figure 6B). The change in ABTS radical scavenging activity was generally consistent with that of DPPH, with all groups showing a gradual decline during storage. Both the 2.5C and 4C treatments maintained significantly higher ABTS scavenging capacity, with the 2.5C treatment performing best (Figure 6C). The enhanced DPPH and ABTS radical scavenging capacities further demonstrate that MAP treatments preserve higher antioxidant capacity in broccoli [9,13]. These findings suggest that MAP treatment helps delay the depletion of antioxidant substances and enhances the free radical scavenging capacity of broccoli.

**Figure 7:**
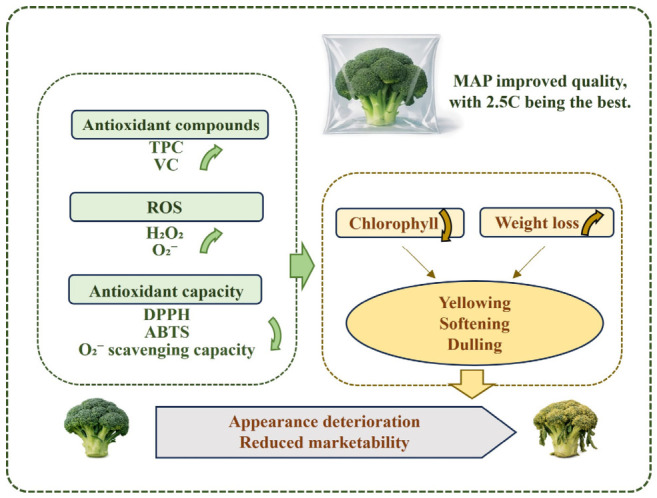
Proposed physiological mechanism to delay senescence of broccoli by modified atmosphere packaging treatments during storage. Thick curved arrows pointing upward and downward indicate positive and negative correlations, respectively.

Imbalanced ROS metabolism is one of the major factors to induce postharvest senescence and quality deterioration in broccoli [50]. Excessive accumulation of ROS such as O_2_•^−^ and H_2_O_2_ can induce membrane lipid peroxidation, chlorophyll degradation, and cellular structural damage [9,51,52,53]. Both MAP treatments effectively delayed senescence of broccoli florets, with the 2.5C treatment showing the most pronounced effect. MAP treatments enhanced O_2_•^−^ scavenging rates and better maintained DPPH and ABTS radical scavenging capacities, indicating that these treatments help strengthen ROS-scavenging capacity and maintain redox homeostasis. The relatively high levels of Vc and total phenolics, together with stronger free radical scavenging capacity, collectively indicate that MAP helps preserve the stability of the postharvest antioxidant capacity in broccoli, thereby delaying quality deterioration (Figure 7). This interpretation is further supported by the correlation analysis shown in Figure S1, in which antioxidant-related indicators were closely associated with ROS accumulation and quality-related parameters, suggesting that the maintenance of antioxidant capacity was related to delayed chlorophyll degradation and senescence in broccoli.

## 4. Conclusions

In the present study, the postharvest preservation of broccoli packed in polyethylene bags modified with nanomaterials at different thicknesses of 25 μm and 40 μm (referred to as 2.5C and 4C, respectively) was investigated at the physiological and biochemical levels. Compared with the control, both the 2.5C and 4C MAP treatments remarkably delayed floret senescence by inhibiting the rapid color change from green to yellow and suppressing chlorophyll degradation, with the 2.5C treatment showing a more pronounced effect during storage. MAP treatments helped maintain commercial quality by preserving total phenols and Vc content, retaining stem firmness and surface glossiness, regulating respiration rate and reducing water loss. The treatments also effectively suppressed the accumulation of O_2_•^−^ and H_2_O_2_. Furthermore, the radical scavenging capacities indicated by DPPH and ABTS assays, together with the O_2_•^−^ scavenging rate, demonstrated that MAP treatments enhanced free radical scavenging capacity in broccoli.

In addition, the preservation effect of nanomaterial-MAP bags may vary with thickness, as different thicknesses alter the atmospheric gas concentration through their different gas permeability. From a physiological perspective, an excessively thick film such as 4C slows down gas exchange to delay yellowing of broccoli, but also may create near-anaerobic conditions inside the package. Such conditions may induce fermentative stress in broccoli tissues during the late storage period. In contrast, the nanomaterial-MAP bag with 2.5C provides a more favorable modified atmosphere by reducing metabolic activity while avoiding excessive gas limitation, which may explain its better performance in maintaining broccoli quality during storage. Accordingly, MAP treatment with an appropriate thickness (i.e., 2.5C) effectively inhibited the decline of Vc and total phenol levels and retarded excessive ROS production. This maintained antioxidant capacity by mitigating the reduction in radical scavenging capacity, thereby further delaying floret chlorophyll degradation and senescence in broccoli. These findings lay a foundation for developing effective and green preservation strategies using physical- and nanomaterial-MAP treatments for fresh broccoli.

## Data Availability

The original contributions presented in this study are included in the article/supplementary material. Further inquiries can be directed to the corresponding authors.

## Supplementary Materials

The following supporting information can be downloaded at: https://www.mdpi.com/article/10.3390/foods15132251/s1, Figure S1: Correlation analysis among different indicators. Pearson correlation coefficients were used to assess the relationships among different physiological and quality-related indicators. The upper triangle shows the correlation coefficient (r) values, while the lower triangle displays the correlations using colored ellipses. Red indicates positive correlations, and green indicates negative correlations. The color scale ranges from −1 to 1. MAP with 25 μm and 40 μm thickness were applied. Data are presented as mean ± standard deviation. Asterisks indicate significant differences at *p* < 0.05 and <0.01.
