# Supplementary material for: Modified Atmosphere Packaging Delays Senescence and Chlorophyll Degradation by Enhancing Antioxidant Capacity in Postharvest Broccoli
Source: Foods. 2026 Jun 23;15(13):2251. doi: 10.3390/foods15132251 (PMC13360619; doi:10.3390/foods15132251)
Supplement: Supplementary file 1 [file foods-15-02251-s001.zip › foods-4342326-supplementary.pdf]

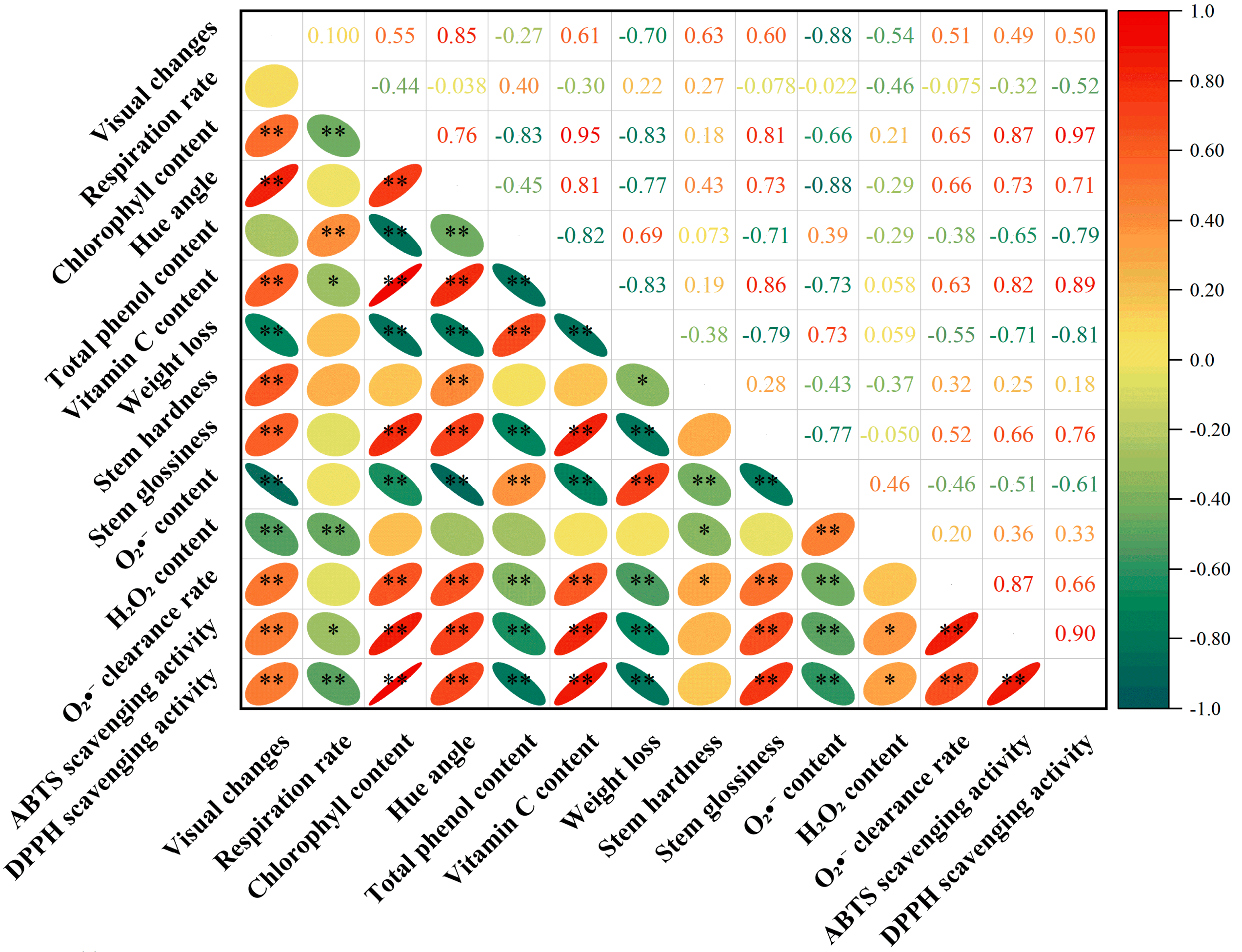

* p<=0.05 ** p<=0.01

Figure S1. Correlation analysis among different indicators. Pearson correlation coefficients were used to assess the relationships among different physiological and quality-related indicators. The upper triangle shows the correlation coefficient (r) values, while the lower triangle displays the correlations using colored ellipses. Red indicates positive correlations, and green indicates negative correlations. The color scale ranges from −1 to 1. MAP with 25 μm and 40 μm thickness were applied. Data are presented as mean ± standard de-viation. Asterisks indicate significant differences at P < 0.05 and < 0.01.
